# Three-Dimensional Landscape Pattern Characteristics of Land Function Zones and Their Influence on PM_2.5_ Based on LUR Model in the Central Urban Area of Nanchang City, China

**DOI:** 10.3390/ijerph191811696

**Published:** 2022-09-16

**Authors:** Wenbo Chen, Fuqing Zhang, Saiwei Luo, Taojie Lu, Jiao Zheng, Lei He

**Affiliations:** 1 East China University of Technology, Nanchang 330013, China; 2 Key Laboratory of Mine Environmental Monitoring and Improving around Poyang Lake, Ministry of Natural Resources, Nanchang 330013, China; 3 The Key Laboratory of Landscape and Environment, Jiangxi Agricultural University, Nanchang 330045, China; 4 School of Tourism and Urban Management, Jiangxi University of Finance and Economics, Nanchang 330013, China

**Keywords:** three-dimensional landscape, landscape pattern, land function zone, PM_2.5_

## Abstract

China’s rapid urbanization and industrialization process has triggered serious air pollution. As a main air pollutant, PM_2.5_ is affected not only by meteorological conditions, but also by land use in urban area. The impacts of urban landscape on PM_2.5_ become more complicated from a three-dimensional (3D) and land function zone point of view. Taking the urban area of Nanchang city, China, as a case and, on the basis of the identification of urban land function zones, this study firstly constructed a three-dimensional landscape index system to express the characteristics of 3D landscape pattern. Then, the land-use regression (LUR) model was applied to simulate PM_2.5_ distribution with high precision, and a geographically weighted regression model was established. The results are as follows: (1) the constructed 3D landscape indices could reflect the 3D characteristics of urban landscape, and the overall 3D landscape indices of different urban land function zones were significantly different; (2) the effects of 3D landscape spatial pattern on PM_2.5_ varied significantly with land function zone type; (3) the effects of 3D characteristics of landscapes on PM_2.5_ in different land function zones are expressed in different ways and exhibit a significant spatial heterogeneity. This study provides a new idea for reducing air pollution by optimizing the urban landscape pattern.

## 1. Introduction

Since the reform and opening up policy was established, China’s urbanization rate has maintained rapid growth, from 17.92% in 1978 to 59.58% in 2018, and it is expected to reach 70% by 2035 [1]. The rapid urbanization has led to dramatic changes in land use/land cover. A large number of natural land-use regions have been replaced by artificial landscapes in cities, resulting in a series of environmental problems. In particular, the severe haze pollution that has continued to erupt in many cities of China in recent years has aroused great concern [2,3,4]. PM_2.5_ is considered to be the main pollutant causing haze. It is easy to accumulate toxic and harmful substances to enter the bronchial and alveolar network of the human body through the respiratory route, penetrating into the blood and causing diseases in the internal respiratory tract and cardiovascular system of the human body [5,6]. It can also damage human cardiopulmonary function and even affect the health of the nervous system [7].

In the urban environment, activities such as industrial production and traffic transportation can create PM_2.5_ pollution [8,9]. Moreover, since all human activity is concentrated in a small built-up area with dense population, people are more vulnerable to PM_2.5_ propagation [9]. Therefore, exploring the spatial distribution of PM_2.5_ and its influencing factors is of great significance for controlling urban air pollution and protecting urban public health.

Numerous studies have shown that PM_2.5_ concentration and distribution at the regional scale are mainly affected by both meteorological condition and land use [10,11,12,13]. Compared to the research on the influence of meteorological condition, the research of the impacts of land use on PM_2.5_ is relatively weak and limited. At the regional scale, research on the effects of land use on PM_2.5_ has always focused on land-use types and land-use patterns [14,15,16,17]. Studies based on land-use types often concluded that PM_2.5_ concentrations in construction land were high, while PM_2.5_ concentrations in non-construction land such as forest land were low [18,19], which can give little guidance for land-use pattern optimization. How to express “land use” in urban environments is of great significance to solving this problem. Land function zone is viewed as the limited area possessing an obvious urban function dominated by one land-use type. It is defined from the land function rather than the land-use type point of view [20]. Our research showed that, in urban center areas, land function zone rather than land-use type can affect PM_2.5_ more visibly from the land use point of view [20]. Moreover, current studies on the impact of landscape pattern on PM_2.5_ are mostly on the two-dimensional level. With the rapid process of urbanization, cities in China continue to expand vertically, and three-dimensional (3D) features become more and more obvious. The 3D urban landscape patterns and building forms are crucial to better understand the effects of land use on PM_2.5_, which require further in-depth study [21,22].

Spatial precise simulation of PM_2.5_ is a precondition to conduct research on the impact of land use on PM_2.5_ pollution. However, gaining enough PM_2.5_ data creates a big challenge. Several approaches have been developed over the last decade to solve this problem, including spatial interpolation, air dispersion model, and land-use regression (LUR) model. The spatial interpolation method should be based on dense monitoring sites. However, in reality, the monitoring sites are often too sparse to meet the needs due to the limited local budget. Dispersion models are often infeasible at a high spatial resolution and are extremely dependent on accurate input data [23]. The LUR model is a statistical regression model based on a geographical information system (GIS) platform. It can be used to predict the concentration of atmospheric pollutants at a given site by establishing a statistical relationship between pollutant and predictor variables, e.g., land use, traffic, and physical characteristics. In recent years, the LUR model has been proven to be a valid and cost-effective approach [23].

Using a 3D landscape index to characterize the urban 3D landscape pattern is a common method used in landscape ecology. Because 3D features are the overall attributes of the whole landscape, 3D landscape indices always focus on landscape levels. For example, landscape height indices are established on the basis of building height, while landscape volume indices are calculated on the basis of building volume per unit area. The landscape height index, such as the average building height and staggered degree, can better reflect the overall height characteristics of a certain landscape and the degree of difference [24,25]. Landscape volume index, such as average building volume and congestion, can reflect the crowded and undulation characteristics of the landscape in 3D space [26,27,28]. At present, studies on the effects of 3D landscape pattern on atmospheric environment mainly focus on the influence of 3D pattern on urban heat island effects, local climate change, and pollutant transportation [29,30,31]. The effect of 3D landscape pattern on the distribution of PM_2.5_ is rarely studied.

Taking the central urban area of Nanchang as an example, we first established a system of 3D landscape indices containing landscape height, congestion, undulation, and diversity for further analysis. Then, four types of land function zone (commercial, residential, industrial, and educational) were identified following the Regulations for the Evaluation of the Intensive Use of Construction Land (TD/T 1018-2008), and 3D landscape indices were calculated to illustrate the landscape pattern characteristics of each land function zone. We applied analysis of variance and multiple comparisons to analyze how the 3D landscape indices of different type of function zone differ from each other. Lastly, the land-use regression (LUR) model [32,33,34] was used to simulate the spatial variation of PM_2.5_ concentration, and correlation analysis and geographic weighted regression (GWR) models were used to study the impact of urban 3D pattern on PM_2.5_ distribution and its spatial heterogeneity. This study aims to explore the impact of the 3D urban landscape pattern on the distribution of PM_2.5_ according to PM_2.5_ concentration simulations using the LUR model. This research can be helpful to provide a theoretical and methodological basis for optimizing the urban landscape pattern to alleviate PM_2.5_ pollution.

## 2. Date and Methods

### 2.1. Study Area

**Figure 1:**
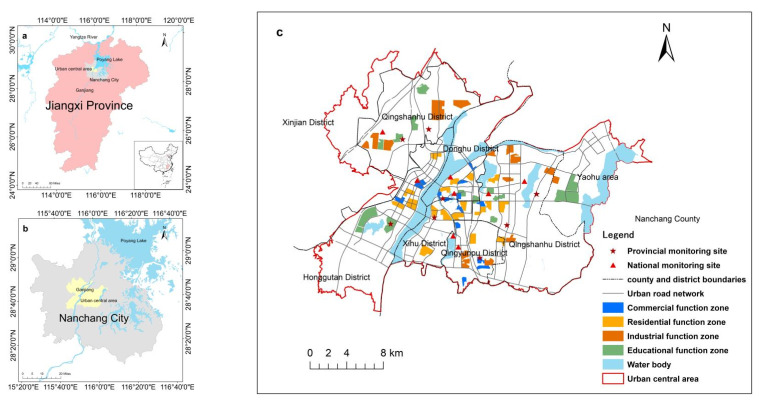
Location of the study area: (**a**) location of Jiangxi Province in China; (**b**) location of urban central area; (**c**) monitoring sites and land function zones in the study area.

Nanchang is the capital city of Jiangxi province (Figure 1) and is one of the core cities in the middle reaches of the Yangtze River. It is located within 28°10′–29°11′ N and 115°27′–116°35′ E on the southwest bank of Poyang Lake, the largest freshwater lake in China. Ganjiang River passes through the city. It is characterized by a subtropical monsoon climate with abundant heat, rainfall, and light. The whole territory of Nanchang is dominated by plains, where the southeastern region is relatively flat and the northwestern is relatively hilly. The city covers six districts and three counties with a total area of 7402.36 km^2^. The study area is the central urban area defined by the land-use master plan, with an area of 562.46 km^2^. The Nanchang Meteorological Bureau has established nine national air quality automatic monitoring sites and 14 provincial ones for monitoring main atmospheric pollutants such as PM_2.5_, SO_2_, CO, and NO_x_ day and night, in which 16 monitoring sites are covered within the study area. In recent years, as with other cities in China, the urbanization and industrialization process of Nanchang has been accelerating, and the urbanization level has been increasing. However, many environment problems, especially air pollution, have become more and more serious.

### 2.2. Data Sources and Methods

#### 2.2.1. Data Sources

The air pollution data used in this study came from the daily average concentration of PM_2.5_ monitored by the 16 national and provincial automatic air quality monitoring sites covered by the central urban area of Nanchang city in 2019. The meteorological data for 2019 came from the China Meteorological Science Data Sharing Service Network. The relevant land-use data was taken from the urban cadastral map of Nanchang City. The 3D data came from Baidu’s real-world map, Nanchang’s 3D map, combined with field survey data.

#### 2.2.2. Identification of Urban Land-Use Function Zones

The land function zone is viewed as the limited area possessing an obvious urban function dominated by one land-use type. It is defined from the land function rather than land-use type point of view [20]. In this study, land-use function zones were identified referring to the Regulations for the Evaluation of the Intensive Use of Construction Land (TD/T 1018-2008). The area proportion of the dominant land-use type is regulated, i.e., residential, commercial, and educational land-use types in their corresponding function zone must account for more than 50%, and industrial land use in the industrial function zone must account for more than 40%. Four types of function zone, i.e., residence, industry, education, and commerce, were identified in the study area. Only the areas with distinct land-use function were identified and used in this study. Other areas with mixed land-use functions were not used for sake of analyzing the impacts on PM_2.5_ with high accuracy. After considering the urban master plan, land-use master plan, and urban function zone identification through an investigation, the land function zones in the urban central area were identified as shown in Figure 1, covering nearly 1% of the study area.

#### 2.2.3. The 3D Landscape Indices Adopted

Many studies have applied a 3D landscape index to quantify urban building morphology. Commonly, a 3D landscape index is defined in terms of height, congestion, and fluctuation (Table 1). For example, average building height, average volume density, landscape spatial dispersion, and landscape fluctuation are most frequently used in the study of 3D landscape patterns (Table 2). They can better represent the landscape height, congestion, and fluctuation. However, the diversity of landscape is also the core manifestation of landscape heterogeneity. In this study, two indices of building diversity and building uniformity were supplemented to express the equilibrium degree of the diversity and distribution of buildings of different height categories. Together with commonly used landscape indices, a more complete 3D landscape pattern index system was formed to characterize 3D spatial features (Table 1 and Table 2).

For the calculation of the 3D landscape index, we obtained the number of floors in each function zone according to Baidu’s real-world map, Nanchang’s 3D map, and a field survey. With reference to the building construction standards, the height of each building in all function zones was estimated in accordance with the standard house floor height of 2.8 m and plant floor height of 5.0 m. According to the regulation of Residential Design Code (GB 50096-2011) and Design of Civil Buildings (GB 50352-2005), the architectural landscape was divided into eight categories, namely, bungalows (2.8–5.0 m), low-rise (5.6–10.0 m), multistory (11.2–15.0 m), and high-rise (19.6–28.0 m), high-rise one (30.8–50.4 m), high-rise two (53.2–70.0 m), high-rise three (73.2–98.0 m), and super high-rise (>100 m). The volume of the building was equal to the product of the contour area of the top floor of the building and the building height. The top contour of the building was obtained through remote sensing using a combination of area-based object classification and artificial interpretation.

#### 2.2.4. LUR Modeling

The LUR model, i.e., land-use regression model, is a commonly used method to simulate the spatial and temporal differentiation of urban air pollution [35], and many researchers have conducted productive studies using this model [35,36]. The theoretical basis of this method is that the spatial distribution of atmospheric pollutants is related to geospatial factors such as land use. Regression equations are constructed through the pollutant data monitored by ground monitoring sites and the surrounding geospatial elements to predict the concentration of pollutants in other regions [37,38]. The regression equation y = β_0_ + β_1_ × 1 + … + β_i_x_i_ is gained as a map by multiplying all cells in the contributing variable layers (x_1_, …, i) by their associated coefficients (β_1_, …, i) with the constant intercept β0.

According to the research of Yang Haiou [20], six buffers (500 m, 1000 m, 1500 m, 2000 m, 3000 m, 4000 m, and 5000 m) were built around the 16 monitoring sites in the central urban area of Nanchang City. The buffers were overlaid and analyzed with the urban cadastral data and road networks, and the predictive variables in each buffer were extracted. According to the relevant geographical variables commonly used in the construction of the LUR model, the population, traffic factors, land use, and meteorological factors were selected as the independent variables of the models (Table 3).

#### 2.2.5. Impact of 3D Landscape Pattern on PM_2.5_

The average value of PM_2.5_ in residential, commercial, industrial, and educational function zones was calculated in this study. Correlation was analyzed between PM_2.5_ in the functional zone and the 3D landscape index. Traditionally, statistics-based nonspatial global models, such as the linear homeopathic model and exponential model, were used for discovering how the landscape pattern can affect PM_2.5_ [39,40]. In fact, landscape pattern’s effects on PM_2.5_ usually present with spatial heterogeneity on local scale. Even for the same landscape, the responses of PM_2.5_ to the different arrangement layouts will be different. Hence, the local spatial model can more appropriately reflect the coupling relationship between them [35,37]. Compared to statistics-based nonspatial global models, GWR considers the local effects of spatial objects and has a higher degree of advantage for exploring the coupling relationship between pattern and process at the local spatial scale [38]. The model formula is as follows:

(1) $$y_{i}=\beta_{0}(u_{i},v_{i})+\overset{m}{\underset{k=1}{\sum }}\left(u_{i},v_{i}\right)x_{\mathrm{ik}}+\varepsilon_{i},$$

where (u_i_, v_i_) is the coordinate of sampling point i, and β_k_ (u_i_, v_i_) is the k-th regression coefficient at sampling point i. In order to further explore the influence mode and degree of landscape spatial pattern on PM_2.5_ concentration, a geographic weighted regression model was used to couple the spatial pattern with PM_2.5_ concentration. Considering that there may be a problem of collinearity between the 3D landscape indices, only one index in each feature with the strongest correlation with PM_2.5_ participated the model construction. The standardized residuals of the geographic weighted regression model were verified using Moran’s I index. A *p*-value result greater than 0.05 indicates that the standardized residuals of the model were discrete, and the model results were credible.

## 3. Results

### 3.1. Distribution of Land-Use Function Zones

Figure 1 shows the land-use function zones identified in Nanchang City. The residential function zone and commercial function zone were closely distributed, mainly concentrated in Honggutan District, a newly developed area of Nanchang City, Donghu District, the center of the old city, and Qingyunpu District. The commercial function zones were mainly distributed in a strip shape along with the residential function zones. The industrial function zones and educational function zones were mainly distributed around the residential and commercial function zones. The industrial function zones were mainly located in the south of city, where various development zones were established for the development of industry. Educational function zones were mainly distributed in Honggutan District and Yaohu area, where the universities and research institutes were concentrated. The statistical features of the function zones are shown in Table 4.

### 3.2. The 3D Characteristics of Land-Use Function Zone

The 3D landscape indices of the land function zone were calculated. Then, we used one-way ANOVA and LSD post-hoc multiple comparison analysis methods to analyze if there were significant differences in the 3D characteristics in different function zones (Table 5 and Table 6).

It can be seen from the above tables that the industrial function zone was significantly different from the other function zones. The educational function zone was significantly different from the residential and commercial function zones in landscape volume density, but not significantly different from the industrial function zone. It differed from the residential, commercial, and industrial function zones in landscape spatial variation and architectural diversity. In addition, the educational function zone was not significantly different from the commercial function zone in terms of building uniformity, but it was different from the industrial and the residential function zones in terms of varying degrees. There was a significant difference in building uniformity between the residential and commercial function zones. It can be seen that the 3D landscape indices constructed could better reflect the 3D characteristics and differences in different land function zones.

### 3.3. Spatial Heterogeneity of PM_*2.5*_ Using LUR Model

We used the monthly average value, i.e., panel data from 16 monitoring sites, as the dependent variable for LUR modeling. Accordingly, a total of 16 × 12 = 192 groups of data were used for the model construction, in which three-quarters were randomly selected for modeling, and the remainder were used for cross-validation. The model-building algorithm adopted in this study was proposed in accordance with Henderson et al. [38]. Firstly, we calculated the correlation of the respective variables with PM_2.5_, selected the highest ranked variable in each subcategory, and discarded the subcategory with a correlation coefficient greater than 0.6 for that variable. Secondly, all remaining variables were used to construct multiple stepwise linear regression equations, where the variables that did not have a significant *t*-statistic at a 90% confidence level were removed. The entire process was repeated until the convergence was obtained, and the optimal model was regarded as the final model. The prediction errors of all models were tested by comparing the predicted and measured values with the average absolute error rate and RMSE.

**Figure 2:**
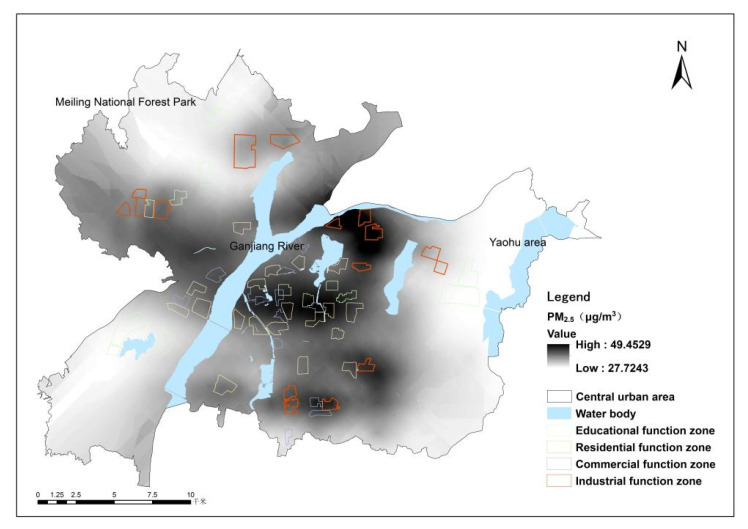
Spatial distribution of PM_2.5_ concentration.

The LUR model of PM_2.5_ was finally built (Table 7). Only four independent variables, i.e., XVEG5000, XPRE, XPRS_Sea, and XINDU500, were significant in the constructed model. It can be seen that the adjusted *R*^2^ value of the model was 0.917, indicating that the constructed model had good adaptability and strong ability to explain the spatial variation. In addition, the average absolute error rate and the root-mean-square error (RMSE) indicated that the model had higher verification accuracy. In terms of land use, we can also see from Table 7. that ecological land and industry land distribution had significant effects on the spatial distribution of PM_2.5_. The simulation results of the spatial distribution of PM_2.5_ are shown in Figure 2. They indicate that, in general, the distribution of PM_2.5_ in the study showed obvious spatial heterogeneity, and the concentration of PM_2.5_ gradually decreased from the central area to the periphery. Together with the spatial distribution of land use, we can see that high-PM_2.5_ areas occurred in the industrial zones in the east, northwest, and south, where the industrial parks of Nanchang city were concentrated. Low-PM_2.5_ areas were distributed along Ganjiang river and in the Meiling National Nature Reserve Park densely populated with forest. However, how land use affects PM_2.5_ from the land function zone point of view still needs deeper studies.

### 3.4. Inference of 3D Indices on PM_2.5_ in Different Land Function Zones

On the basis of the simulation results of PM_2.5_ distribution, we calculated the average PM_2.5_ concentration in each function zone of residence, commerce, industry, and education, and we analyzed the correlation between PM_2.5_ and the 3D landscape index. The correlation coefficients are shown in Table 8.

In the industrial function zone, all 3D landscape indices were positively correlated with the concentration of PM_2.5_. The landscape height density, building diversity, and building uniformity were significantly positively correlated with PM_2.5_ concentration at the 0.05 significance level. This indicated that the average height and distribution of buildings in industrial function zone had a significant effect on the concentration of PM_2.5_. In the educational and residential function zones, the landscape height density and PM_2.5_ concentration were significantly negatively correlated (*p* < 0.05), indicating that the building height had an important effect on PM_2.5_ concentration. In the commercial function zone, all 3D landscape indices were negatively correlated with the concentration of PM_2.5_. Among them, building uniformity, building diversity, and landscape spatial variation were significantly negatively correlated with PM_2.5_ concentration (*p* < 0.05).

### 3.5. The Spatial Variance of 3D Indices Using GWR Model

In order to improve the modeling accuracy and remove the collinear effects of 3D landscape indices, we chose one index with the strongest correlation and another with a correlation coefficient less than 0.6 for each type of land function zone as independent variables to participate in model construction according to the independent variable screening method proposed in Section 3.2. That is, the industrial function zone selected landscape diversity and landscape unevenness, the educational and residential function zones selected landscape height density and building uniformity, and the commercial function zone selected landscape spatial dispersion and building uniformity for GWR modeling [41,42,43]. Table 9 shows the fitting and testing results of the GWR model of the four function zones.

The adjusted *R*^2^ values of the models of the industrial and commercial function zones were all above 50%, indicating the model performed well and could better explain the relationship between the independent variable and PM_2.5_ concentration. The adjusted *R*^2^ value of the residential function zone was low, and the model fitting effect was limited. According to the results of Moran’s I verification, the *p*-values of the four models were less than 0.5, which indicated that the standardized residuals were in a random distribution state and the model results were credible.

**Figure 3:**
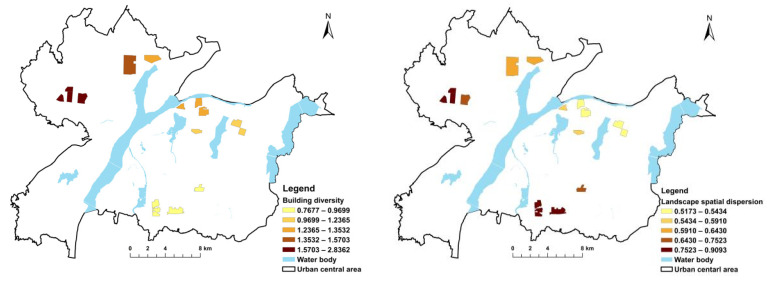
GWR results for industrial function zone.

**Figure 4:**
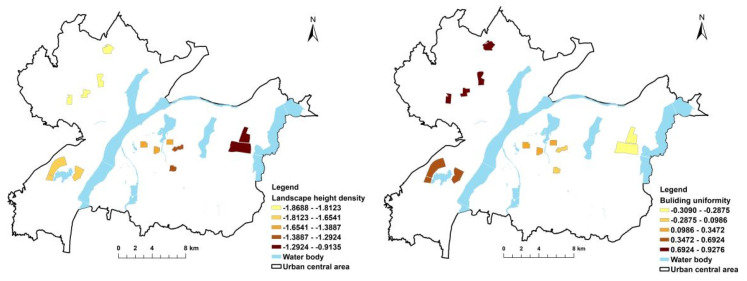
GWR results for educational function zone.

**Figure 5:**
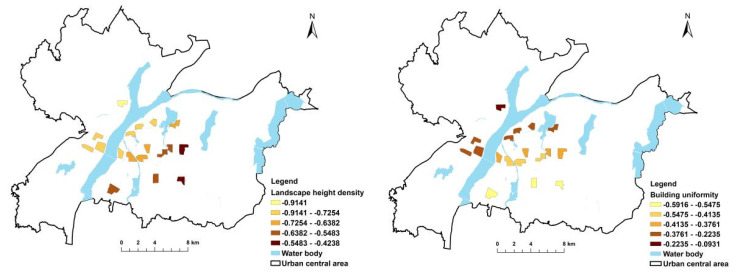
GWR results for residential function zone.

**Figure 6:**
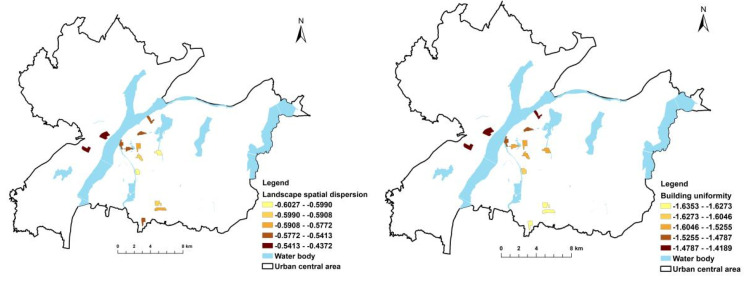
GWR results for commercial function zone.

Figure 3, Figure 4, Figure 5 and Figure 6 show the independent variable regression coefficients of the GWR model for each function zone. In the industrial function zone, the regression coefficients of building diversity and landscape spatial dispersion were generally positive, indicating that the PM_2.5_ concentration increased with the increase in the type of buildings and the degree of dispersion. In the education function zone, the landscape height density had a negative effect on PM_2.5_ concentration, while the building uniformity mainly had a positive effect on PM_2.5_ concentration, and the impact intensity gradually decreased from west to east. In the residential function zone, the regression coefficients of both the landscape height density and the building uniformity were negative, indicating that they had a negative effect on the PM_2.5_ concentration. This also demonstrated that, in the central area of the city with higher building density and higher building height, the PM_2.5_ concentration decreased with the increase in average building height and the increase in building uniformity level. In the commercial function zone, the regression coefficients of landscape spatial variation and building uniformity were also negative, and the absolute value of the regression coefficient gradually increased from north to south. It can be seen that the landscape spatial dispersion and building uniformity had a negative effect on PM_2.5_ concentration, and the intensity of the effect gradually increased from north to south. In addition, in the commercial function zone, the absolute value of the regression coefficient of the building uniformity was significantly higher than the building uniformity, which indicated that the degree of influence of the building uniformity PM_2.5_ concentration was higher than that of the landscape space.

## 4. Discussion

Many studies discovered that PM_2.5_ concentration and distribution at the regional scale were mainly affected by both meteorological condition and land use. Compared to studies on meteorological condition, the research of the impacts of land use on PM_2.5_ was relatively limited, especially in urban areas. How to express “land use” in an urban environment is of great significance to solving this problem. At present, studies on the effects of land use on PM_2.5_ always focus on land-use type. The results often concluded that PM_2.5_ concentrations in construction land were high, while PM_2.5_ concentrations in non-construction land such as forest land were low, which can provide little guidance for land-use optimization, especially for urban land use. In this study, we put forward the idea of land function zone instead of land-use type to analyze the relationship between land use and PM_2.5_. It is expected that this new and innovative approach will deepen understanding of the coupling relation between land use and PM_2.5_ in urban area. Furthermore, with the development of the urbanization process, city landscape 3D characteristics became more and more obvious. Studies have shown that the 3D index was more descriptive than the two-dimensional index, and it could better reflect the complexity of the city [24]. On the basis of the existing 3D landscape index, a 3D landscape index system with six indices reflecting the height, congestion, fluctuation, and diversity characteristics of the urban land use was constructed for analyzing the 3D spatial pattern of the central area in Nanchang city based on land function zone. Some studies used the arc–chord ratio rugosity index (ACR) to quantify the complexity of the 3D landscape structure [44]. Considering the difficulty of obtaining data, these indices were not used in this study.

It was found that the 3D landscape indices of industrial and educational function zones were significantly different from those of the other types of function zones, i.e., residential function zone and commercial function zone. The results of this study were consistent with the actual situation of the city. The land function zone of a city refers to a certain type of social economic activity at the dominant space, and it plays an important role in the urban economic and social functions [45]. Due to the high population density, high rents, and huge benefits driven by residential and commercial function zone, the space will become more and more crowded. The industrial function zones are mainly distributed on the periphery of the city, and the land rent and space congestion are much lower than those in the city center. The educational function zone is mainly composed of independent universities and research institutions, with dense buildings among the industrial, residential, and commercial function zones. Thus, different land function zones in the city will present different 3D characteristics. Therefore, the constructed 3D landscape index system can accurately distinguish the differences among them. Overall, the 3D landscape index system constructed in this study can better reflect the spatial heterogeneity of the 3D landscape features of different function zones in Nanchang city.

Previous studies all agreed that the number of monitoring sites did have an important impact on the results of LUR modeling; however, in reality, the monitoring sites are often too sparse to meet the needs due to the limited local budget. At present, the LUR model is generally recognized as an economic and effective method for simulating the concentration of air pollutants in urban areas [46,47,48]. However, a consensus has not been reached on how many monitoring sites are required for this method. Many studies believed that the number of sites should be determined in combination with the population and scale of the city (Hoek, et al., 2008). In this paper, only the data from 16 monitoring sites were used for LUR modeling, which is really a limitation of our study. Although the number of sites was not large, there was one site for every 35 km^2^. Compared to previous studies, the density of sites in this study was the same or even higher [20,37,38]. Furthermore, the monthly average concentration data, i.e., panel data, were used for modeling, indicating that a total of 16 × 12 = 192 groups of data were used for the model construction, in which three-quarters were used for modeling and the remainder were used for verification. This could supplement the limitation of monitoring sites in some cases. Nevertheless, supplementing the monitoring sites requires extensive costly labor and material inputs which are difficult to achieve at present, but this could represent a research direction to improve precision of the study in the future.

In this study, the traffic variables were not significant in modeling. This is in contrast to the conclusion of previous studies [35,49,50]. One possible reason is that the study area is located in the central area of Nanchang City, mostly covered by built-up areas with a complete transportation system, and the road density around monitoring sites did not significantly differ. Accordingly, it did not significantly affect the heterogeneity of PM_2.5_ distribution, but this does not necessarily mean that traffic variables do not affect PM_2.5_ concentration. Another reason could be that we used monthly average PM_2.5_ data rather than daily data, whose difference was averaged, and the spatial heterogeneity was depressed.

In previous studies, the adjusted *R*^2^ value of the LUR model was mostly less than 0.8 [37,51,52,53]. The adjusted *R*^2^ value of the LUR model constructed in this study reached 0.9, indicating that the model performed well in explaining the spatial variability of PM_2.5_ concentrations. Early studies focused on analyzing the impact of single building and block changes on air pollution, and they rarely analyzed the impact of 3D landscape spatial pattern on air pollutant [54]. This study found that, in the industrial function zone, both building diversity and landscape spatial dispersion had a positive effect on PM_2.5_, of which building diversity had a greater impact. Considering that the types of buildings in the industrial function zone were relatively simple and the average height was low, when the height and type of buildings increased, the pollutant emissions in the industrial zone increased, and the PM_2.5_ concentration increased accordingly. This conclusion is consistent with industrial pollution and emissions being the main sources of PM_2.5_ pollution [15,55]. Landscape height density and building uniformity had a negative effect on PM_2.5_ concentration in the residential and commercial function zones, which indicated that the increase in the height of some buildings and the improvement of uniformity were beneficial to the diffusion of PM_2.5_. This result is similar to the research of urban canopy rugosity on pollution diffusion [56]. The correlation between rugosity and wind speed changed with the height of the building. When the building height was higher than a certain critical value, it was negatively correlated with the wind speed, whereas, when it was below the critical value, it was positively correlated with the wind speed [56]. In addition, changes in building height had an impact on the storage and release of heat, which also indirectly affected the photochemical ability and diffusion of pollutants [56].

## 5. Conclusions

This paper firstly constructed a 3D landscape index system from the 3D perspective of height, congestion, fluctuation, and diversity characteristics. Then, by means of land function zone identification, the 3D pattern difference in different land function zones was analyzed taking Nanchang City’s central urban area as a case. On the basis of the LUR simulation of PM_2.5_, the relationship between 3D characteristics of land function zone and PM_2.5_ was analyzed. Furthermore, a geographic weighted regression model was constructed to explore the heterogeneity of the impact of 3D urban landscape pattern on the distribution of PM_2.5_, yielding the following indications:

(1) The analysis of variance and multiple comparison tests showed that there were significant differences in the overall 3D landscape pattern in different urban land function zone, indicating that the 3D landscape index system constructed can reflect the 3D characteristics of different urban land use.

(2) Correlation analysis results indicated that the impact of landscape spatial pattern on PM_2.5_ concentration distribution varied with land function zone. In the industrial function zone, building diversity and landscape spatial dispersion had a positive effect on PM_2.5_ concentrations. The landscape height density in the educational function zone had a negative effect on PM_2.5_ concentration, while the building uniformity played a positive role. Landscape height density and building uniformity had a negative effect on PM_2.5_ concentration in the residential function zone. In the commercial function zone, landscape spatial dispersion and building uniformity had a negative effect on PM_2.5_ concentration.

(3) In addition, the results of GWR models showed that the 3D characteristics of landscapes in different land-use function zones affect PM_2.5_ concentrations in different ways and degrees, exhibiting significant spatial heterogeneity.

This study can provide some suggestions for city planners to reduce PM_2.5_ pollution by means of optimizing 3D urban landscape pattern from the congestion, fluctuation, and diversity points of view.

## Figures and Tables

**Table 1:** Features and methods of 3D landscape pattern description.

3D Feature	Schematic Diagram	Methods Description
Height	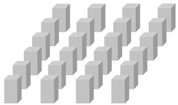 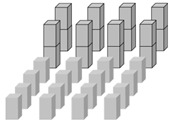	Height is the most basic and intuitive feature that distinguishes 3D space from the 2D plane. It can be reflected by the average value of building height.
Congestion	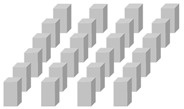 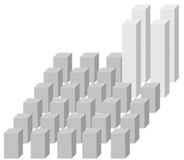	Congestion denotes the density of buildings in the sample area. The different volume, shape, floor area, and peripheral outline of urban buildings will affect the architectural landscape pattern of the area. The openness of buildings plays an important role in atmospheric diffusion. The degree of congestion can be calculated from the ratio of the sum of the building volume and the maximum height of the building multiplied by the area of the sample area.
Fluctuation	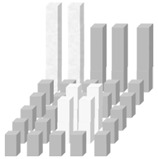	Fluctuation shows the difference in the building height in the sample area. The fluctuation feature is also a basic indicator to describe the 3D feature of the landscape. It can be calculated from the difference between the highest value and the lowest value of the building height in the sample area.
Diversity	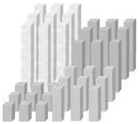 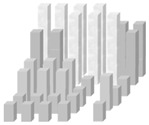	Diversity indicates the number of buildings with different heights in the sample area. The diversity index in the 2D plane is often used to calculate the heterogeneity of the community. In the 3D environment, after dividing the building into different categories according to height, the spatial characteristics can be measured from the spatial heterogeneity. The buildings are divided into different categories according to their height, and the heterogeneity of the architectural landscape can be calculated using the Shannonville diversity index and uniformity index.

**Table 2:** Formulas of 3D landscape indices.

3D Feature	3D Landscape Index	Formula	Description
Height	Landscape height density	$\bar{H}=\frac{\sum_{i=1}^{n}H_{i}}{n}$	Representing the average height of the architectural landscape. H_i_ is the height of building i, and n is the number of buildings in the sample area.
Congestion	Landscape volume density	$E=\frac{\sum_{i=1}^{n}V_{i}}{H_{\max}\times S}$	V_i_ is the volume of building i, $H_{\max}$ is the maximum height of the building in the sample area, and S is the area of the sample area.
Fluctuation	Landscape spatial dispersion	$L=\frac{\sqrt{\frac{1}{n}\sum_{i=0}^{n}{\left(H_{i}-\bar{H}\right)}^{2}}}{\bar{H}}$	Representing the degree of dispersion of building height. H_i_ is the height of building i.
	Landscape fluctuation	$\mathrm{LHR}=H_{\max}-H_{\min}$	$H_{\max}$ is the maximum building height in the landscape, and $H_{\min}$ is the minimum building height in the landscape.
Diversity	Building diversity	$S=-\overset{m}{\underset{i=0}{\sum }}\left(P_{i}\times {\mathrm{lnP}}_{i}\right)$	P_i_ is the percentage of the area occupied by buildings of type i, and m is the total number of building types in the landscape.
	Building uniformity	$E=\frac{S}{S_{\max}}\times 100\%$	Representing the uniformity of building distribution. $S_{\max}$ is the maximum diversity index in the landscape.

**Table 3:** Independent variables of LUR modelling.

Factor	Variable	Description	Unit	Assumed Correlation
Road	MROAD	Proportion of main road length to buffer area	m	+
	SROAD	Proportion of secondary road length to buffer area	m	+
	TAL	Proportion of total road length to buffer area	m	+
Land use	VEG	Proportion of ecological area (forest, water, etc.) to buffer area	%	−
	INDU	Proportion of industrial land area to buffer area	%	+
	WAT	Proportion of water area to buffer area	%	−
	RAR	Proportion of arable land area to buffer area	%	−−−−
Population	POP	Proportion of residential land area to buffer area	%	+
Meteorological factor	PRS	Air pressure	hPa	−−−−
	PRS_Sea	Sea pressure	hPa	−−−−
	WIN	Wind speed	m/s	−−−−
	TEM	Temperature	°C	−−−−
	RHU	Relative humidity	%	−−−−
	PRE_1 h	Hourly precipitation	mm	−−−−

**Table 4:** Statistical features of the function zones.

Type	Number	Max (km^2^)	Min (km^2^)	Mean (km^2^)
Industrial function zone	16	2.88	0.37	0.84
Educational function zone	14	2.93	0.40	1.05
Residential function zone	18	1.13	0.50	0.70
Commercial function zone	13	0.73	0.31	0.39

**Table 5:** Results of one-way ANOVA analysis.

Variable	Type III Sum of Squares	Degree of Freedom	Mean Square	F	Significance
Landscape height density	344.637	3	114.879	5.253	0.003
Landscape volume density	31.167	3	10.389	6.993	0.000
Landscape spatial dispersion	1.085	3	0.362	5.082	0.003
Landscape fluctuation	16,998.259	3	5666.086	11.356	0.000
Building diversity	1.933	3	0.644	4.116	0.010
Building uniformity	1.027	3	0.136	3.894	0.015

**Table 6:** Multiple comparison results of 3D landscape index in land-use function zones.

Variable	Function Zone (I)	Function Zone (J)	Mean Difference	Standard Error	Significance
Landscape height density	Commercial	Residential	−0.996	1.702	0.561
		Educational	−0.362	1.772	0.839
		Industrial	4.818 *	1.746	0.008
	Residential	Commercial	0.996	1.702	0.561
		Educational	0.635	1.635	0.699
		Industrial	5.814 *	1.607	0.001
	Educational	Commercial	0.362	1.772	0.839
		Residential	−0.635	1.635	0.699
		Industrial	5.179 *	1.681	0.003
	Industrial	Commercial	−4.818 *	1.746	0.008
		Residential	−5.814 *	1.607	0.001
		Educational	−5.179 *	1.681	0.003
Landscape volume density	Commercial	Residential	0.378	0.444	0.398
		Educational	1.758 *	0.462	0.000
		Industrial	1.441 *	0.455	0.002
	Residential	Commercial	−0.378	0.444	0.398
		Educational	1.381 *	0.426	0.002
		Industrial	1.063 *	0.419	0.014
	Educational	Commercial	−1.758 *	0.462	0.000
		Residential	−1.381 *	0.426	0.002
		Industrial	−0.318	0.438	0.471
	Industrial	Commercial	−1.441 *	0.455	0.002
		Residential	−1.063 *	0.419	0.014
		Educational	0.318	0.438	0.471
Landscape spatial dispersion	Commercial	Residential	0.017	0.097	0.860
		Educational	0.230 *	0.101	0.027
		Industrial	0.305 *	0.100	0.003
	Residential	Commercial	−0.017	0.097	0.860
		Educational	0.213 *	0.093	0.026
		Industrial	0.288 *	0.092	0.003
	Educational	Commercial	−0.230 *	0.101	0.027
		Residential	−0.213 *	0.093	0.026
		Industrial	0.076	0.096	0.434
	Industrial	Commercial	−0.305 *	0.100	0.003
		Residential	−0.288 *	0.092	0.003
		Educational	−0.076	0.096	0.434
Landscape fluctuation	Commercial	Residential	−2.874	8.130	0.725
		Educational	20.055 *	8.464	0.021
		Industrial	37.428 *	8.341	0.000
	Residential	Commercial	2.874	8.130	0.725
		Educational	22.929 *	7.809	0.005
		Industrial	40.301 *	7.675	0.000
	Educational	Commercial	−20.055 *	8.464	0.021
		Residential	−22.930 *	7.809	0.005
		Industrial	17.372 *	8.028	0.035
	Industrial	Commercial	−37.428 *	8.341	0.000
		Residential	−40.301 *	7.675	0.000
		Educational	−17.372 *	8.028	0.035
Building diversity	Commercial	Residential	0.027	0.144	0.852
		Educational	0.176	0.150	0.246
		Industrial	0.443 *	0.148	0.004
	Residential	Commercial	−0.027	0.144	0.852
		Educational	0.149	0.138	0.287
		Industrial	0.416 *	0.136	0.003
	Educational	Commercial	−0.176	0.150	0.246
		Residential	−0.149	0.138	0.287
		Industrial	0.267	0.142	0.065
	Industrial	Commercial	−0.443 *	0.148	0.004
		Residential	−0.416 *	0.136	0.003
		Educational	−0.267	0.142	0.065
Building uniformity	Commercial	Residential	0.022 *	0.079	0.059
		Educational	0.016	0.082	0.158
		Industrial	0.151 *	0.081	0.003
	Residential	Commercial	−0.022	0.079	0.059
		Educational	−0.006	0.076	0.227
		Industrial	0.129 *	0.075	0.001
	Educational	Commercial	−0.016	0.082	0.158
		Residential	0.006	0.076	0.227
		Industrial	0.135 *	0.078	0.050
	Industrial	Commercial	−0.151	0.081	0.003
		Residential	−0.129 *	0.075	0.001
		Educational	−0.135 *	0.078	0.050

Note: * *p* < 0.05.

**Table 7:** Results of LUR model.

Variable	Land Use Regression Model	Goodness of Fit (*R*^2^)	Adjusted *R*^2^	Average Absolute Error Rate	RMSE
PM_2.5_	Y = 41.308 − 5.921XVEG5000 + 40.316XPRE − 26.102XPRS_Sea + 4.088XINDU500	0.958	0.917	0.094	4.583

VEG5000: proportion of ecological area in 5000 m buffer zone; PRE: hourly precipitation; PRS_Sea: sea-level pressure; INDU500: 500 m proportion of industrial land area in 500 m buffer zone.

**Table 8:** Correlation between 3D landscape index of functional zones and PM_2.5_ concentration.

3D Landscape Index	Industrial Function Zone	Educational Function Zone	Residential Function Zone	Commercial Function Zone
Landscape height density	0.518 *	−0.556 *	−0.546 *	−0.061
Landscape volume density	0.048	0.325	−0.095	−0.270
Landscape spatial dispersion	0.342	0.112	−0.051	−0.574 *
Landscape undulation	0.454	−0.262	−0.263	−0.473
Building diversity	0.589 *	−0.211	−0.304	−0.602 *
Building uniformity	0.482 *	0.108	−0.358	−0.635 *

Note: * *p* < 0.05.

**Table 9:** Results of GWR model.

Variable	Goodness of Fit (*R*^2^)	Adjusted *R*^2^	Moran’s Index	*p*-Value
Industrial function zone	0.8832	0.7145	0.1181	0.3431
Commercial function zone	0.7938	0.6852	0.0123	0.6876
Educational function zone	0.5295	0.4569	−0.2052	0.4625
Residential function zone	0.3129	0.2145	0.1440	0.3091

## Data Availability

The data presented in this study are available on request from the first author.
